# The (cost-) effectiveness of exergaming in people living with dementia and their informal caregivers: protocol for a randomized controlled trial

**DOI:** 10.1186/s12877-019-1062-x

**Published:** 2019-02-19

**Authors:** Joeke van Santen, Rose-Marie Dröes, Judith E. Bosmans, Olivier A. Blanson Henkemans, Sjef van Bommel, Esther Hakvoort, Ronald Valk, Carla Scholten, Joris Wiersinga, Annemieke van Straten, Franka Meiland

**Affiliations:** 1 Department of Psychiatry, Amsterdam UMC, location VUmc, Oldenaller 1, 1081 HJ Amsterdam, the Netherlands; 2 Amsterdam Public Health research institute, Van der Boechorststraat 7, 1081 BT Amsterdam, the Netherlands; 3 Department of Health Sciences, Faculty of Science, Vrije Universiteit Amsterdam, Amsterdam Public Health research institute, De Boelelaan 1081, 1081 HV Amsterdam, the Netherlands; 4 Child Health, TNO, Schipholweg 77, 2316 ZL Leiden, the Netherlands; 5 Sjef van Bommel Management & Support, Stadionweg 53HS, 1077 RZ Amsterdam, the Netherlands; 6 Scientific Committee Evean, Waterlandplein 5, 1441 RP Purmerend, the Netherlands; 7 HilverZorg – Day-care centre Zonnehoeve, Loosdrechtse Bos 9, 1213 RH Hilversum, the Netherlands; 8 Embedded Fitness B.V., Kapelweg 11, 5756 AJ Vlierden, the Netherlands; 9 SilverFit B.V., Edisonweg 7, 3442 AC Woerden, the Netherlands; 10 Department of Clinical- neuro- and developmental Psychology, Faculty of Behaviour and Movement Sciences, VU University Amsterdam, Van der Boechorststraat 7, Amsterdam, the Netherlands; 11 GGZ inGeest Dienst Onderzoek en Innovatie, Oldenaller 1 (room H3.08) Postbus 74077, 1070 BB Amsterdam, The Netherlands

**Keywords:** Randomized controlled trial, Dementia, Exergaming, Physical activity, Physical functioning, Quality of life, Cost effectiveness

## Abstract

**Background:**

Physical activity is linked to benefits such as increased physical fitness, cognition, emotional and social functioning, general health and well-being in older people. Some evidence suggests that this also applies to people living with dementia. However, it can be harder for them to perform physical activities, due to several barriers, such as issues with orientation and balance problems. A relatively new type of physical activity called exergaming may help them overcome these barriers. Exergaming is “physical exercise interactively combined with cognitive stimulation in a gaming environment”. The aim of our study is to evaluate the effectiveness and cost-effectiveness of exergaming compared to regular activities in people living with dementia, who attend day-care centres. Additionally, we want to investigate whether the exergaming activity for the person living with dementia, also (indirectly) affects the informal caregiver, as well as which facilitators and barriers to implementation of exergames for this target group exist.

**Methods:**

A cluster Randomized Controlled Trial (RCT), with economic and process evaluations alongside will be carried out. In the Netherlands, 24 day-care centres are randomized in the experimental or control group. The study group will consist of 224 dyads (community-dwelling participants with dementia and their informal caregivers), who are interviewed at baseline, and at 3 and 6 months of follow-up. The participant with dementia has to visit the day-care centre for at least two days per week, have a diagnosis of mild to moderate dementia and have an informal caregiver present, who is willing to participate. Societal cost data will be collected during interviews, using healthcare utilization diaries, and from day-care centres. The process evaluation will only involve the experimental group, and will include an online survey, qualitative interviews and focus groups.

**Discussion:**

This study will contribute to the evidence base that more effective exercise among people with dementia will result in positive effects on their wellbeing and quality of life. This will motivate people with dementia to be physically active. We also envision that there might be a positive effect on the burden of care experienced by their informal caregivers.

**Trial registration:**

This trial was registered at the Netherlands Trial Register (NTR) on December 10, 2015 (number: NTR5537), this publication is based on protocol amendment number 01, issue date 28 December 2018. This includes all items from the World Health Organization Trial Registration Data Set [see Additional file 1].

**Electronic supplementary material:**

The online version of this article (10.1186/s12877-019-1062-x) contains supplementary material, which is available to authorized users.

## Background

Physical inactivity and a sedentary lifestyle are related to negative health-outcomes, such as increased mortality [1–4], reduced well-being and quality of life [5–7], and an increase in health related costs [8, 9]. On the other hand, physical activity is linked to benefits such as increased physical fitness, cognition, emotional and social functioning, and general health and well-being in older people [10–16]. There is some evidence that this also applies for people living with dementia on outcomes like physical, cognitive, emotional and social functioning [17–26]. However, it can be harder for them to participate in physical activity due to several barriers they may experience. For example, orientation (wandering, getting lost) [27] and balance problems (vertigo, risk of falls) [28] can make it more challenging for people living with dementia to engage safely in physical activities outside the house. Other examples of barriers are a decrease of initiative and interest (increase of apathy) [29, 30], as well as various psychosocial issues (i.e. negative attitudes toward exercise, lack of perceived behaviour control) [31].

A relatively new type of physical activity called exergaming may help people living with dementia overcome barriers they experience with regard to physical activities [32]. Our definition of exergaming is “physical exercise interactively combined with cognitive stimulation in a gaming environment” [33, 34]. Technology registers the movements of the participant, which influence the course of a game on a screen [35]. There are various exergaming applications, such as games that are interactively connected to motion sensing devices (e.g., *“Kinect©”*, “*Wii Fit©”,* and “*PlayStation©*” games with sports). Other applications are interactive walking or cycling, with a treadmill or a stationary bicycle connected to a screen, on which digital video images of the environment are shown. The participants can pick a route and by their walking or cycling pace adjust the speed of the film [33].

Exergaming is expected to be a joyful activity in itself for people with dementia and, thus, a feasible way to motivate them to engage in physical activities in a safe manner [36–39]. International studies which included people living with dementia show some promising effects of exergaming, for example that it can improve physical [40, 41], cognitive [38, 40] and emotional functioning [41]. Small-scale pilots in Dutch psychogeriatric day-care centres and nursing homes also yielded encouraging results [32, 42, 43]. Moreover, exergaming motivated older people with dementia to exercise longer and the videos sometimes evoked memories, which led to mental activation [42]. Additionally, it was relatively easier for staff to get people with dementia involved in physical activities, because they were motivated to exercise [42]. However, more robust research studies are needed to investigate the effects of exergaming in comparison with the physical activities people normally perform in their daily lives [33]. Furthermore, there is also a lack of research into exergaming for people living with dementia on the effects of informal caregivers, as well as on cost-effectiveness and implementation [33].

This article describes the design of a cluster Randomized Controlled Trial (RCT) and process evaluation that intends to contribute to the evidence base for the effect of exergames on physical activity in people with dementia. The aim of our study is to investigate the effectiveness and cost-effectiveness of exergaming compared to regular activities in people living with dementia, who attend day-care centres. Additionally, we want to investigate whether the exergaming activity for the person living with dementia, also (indirectly) affects the informal caregiver, in regard to quality of life, experienced burden and positive care experiences.

We theorize that exergaming has a positive effect on several domains of quality of life of people living with dementia and their informal caregivers [44]. The physical exercise involved in exergaming may promote physical fitness, as well as the physical and emotional functioning of the person living with dementia. Additionally, through the interaction with the game on a screen the cognitive and emotional functioning of the person living with dementia might be influenced positively. Moreover, the social engagement involved in participating in the exergaming activity in a day-care centre (e.g., by cycling together or interacting with spectators) may positively affect social functioning. For informal caregivers, we expect that they will find it reassuring to know that the person with dementia is engaged in a meaningful activity, that they can talk about afterwards. Moreover, the informal caregivers may notice a positive change in the behaviour of the person with dementia, because they have been engaged in a fun activity. This may contribute to positive care experiences of the informal caregivers as well as a reduction in the experienced burden of the informal caregiver. Taking into account all these factors we expect that exergaming will have a positive effect on the quality of life of both the person with dementia and the informal caregiver.

The objectives of our study are to conduct:

1. An effect evaluation to determine whether:
  - persons with dementia in day-care centres with exergaming are more physically active and have improved mobility compared to persons with dementia in day-care centres without exergaming;
  - exergaming has a positive effect on the physical, cognitive, emotional and social functioning and the quality of life of people with dementia. in comparison to regular activities offered in day-care centres;
  - exergaming has an effect on quality of life, experienced burden and positive care experiences of the informal caregivers;
  - exergaming is cost-effective from a societal perspective in comparison to regular activities in day-care centres for people with dementia and their informal caregivers.
2. A process evaluation to determine:
  - whether people with dementia (in the exergaming group) enjoy the exergaming activity and whether this is related to characteristics of the participant or the context;
  - facilitators and barriers to implementation of exergames for community-dwelling people living with dementia;
  - how people living with dementia, informal caregivers, staff of day-care centres and companies developing exergames think we can best apply the exergaming intervention for people with dementia;
  - how well exergaming was executed in the day centres during the RCT and whether care professionals saw benefits in its use.

## Methods

### Study design and setting

We will study the effectiveness and cost-effectiveness of exergaming in a cluster RCT using a randomized controlled block design. All aspects of this trial (design, conduct, and reporting) will adhere to the requirements of the SPIRIT statement (Standard Protocol Items: Recommendations for Interventional Trials) [45, 46] as well as to the Consolidation Standards of Reporting Trials (CONSORT) guidelines [47]. A completed SPIRIT checklist of our trial protocol is available upon request. We will compare people with dementia (and their informal caregivers), who visit day-care centres which offer exergaming to people with dementia (and their informal caregivers), who visit day-care centres that offer regular activities regarding the following outcome variables: physical activity, mobility and physical, cognitive, emotional and social functioning, quality of life of people with dementia and their informal caregivers, experienced burden and positive care experiences of the informal caregivers, and societal costs. The comparator of regular activities at day-care centres without exergaming has been chosen, to ensure that any between-group effect we will find, can be contributed to the exergaming intervention.

Interviews and tests will be scheduled at the start of the study (baseline), after three and after 6 months (two follow up measurements). In total, 24 day-care centres will be recruited. All day-care centres are located in the Netherlands, there are no restrictions to which parts of the country. A list of the study sites will be available upon request. The process analysis will only be carried out in the experimental group.

### Recruitment and randomization day-care centres

**Fig. 1:**
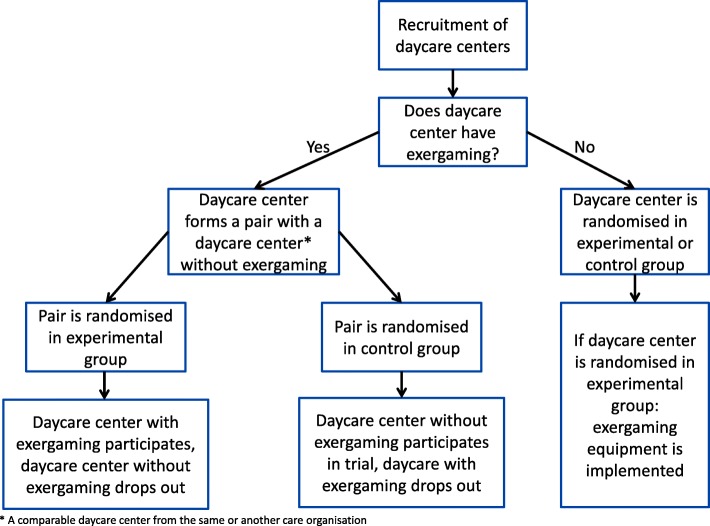
Flow chart of the randomization of day-care centres

An overview of the recruitment and randomization process for the day-care centres can be found in Fig. 1.

We will (if possible) arrange blocks of centres that are similar to one another (in terms of region and/or target group: i.e. severity of dementia, age of onset, ethnicity, group size in the day-care centre). This is to control for environmental factors that might possibly influence the outcomes. Within these blocks, day-care centres will be randomized by an independent researcher, using Random Allocation Software [48], to either the experimental (exergaming) group or control (regular activities) group (12 day-care centres per group). After randomization, the day-care centres will remain in the experimental or control group throughout the duration of the trial.

Day-care centres which already have the required exergaming equipment, will be paired up with a comparable day-care centre from the same or another care organization without the equipment. Then they will be randomized as a pair instead of as an individual day-care centre. If this pair is randomized in the experimental group, the day-care centre with the exergaming equipment will participate in the trial. Similarly, if this pair is randomized in the control group, the day-care centre without the equipment will participate in the trial. Day-care centres which do not have any exergaming equipment yet, can also be enrolled for randomization individually if they are willing and able to buy the exergaming equipment needed for the trial. This has to be confirmed, prior to randomization, and is intended to ensure they can still participate when they are randomized to the experimental group.

Day-care centres in both groups will each receive an allowance of at most € 1.125,-. When they recruit eight persons or more. They can decide themselves how they want to spend this, for example to pay their staff for the extra hours they have to put in to participate in the research project. Day-care centres in the experimental group may also put this amount towards the purchase of the exergaming equipment.

### Participants and setting

**Fig. 2:**
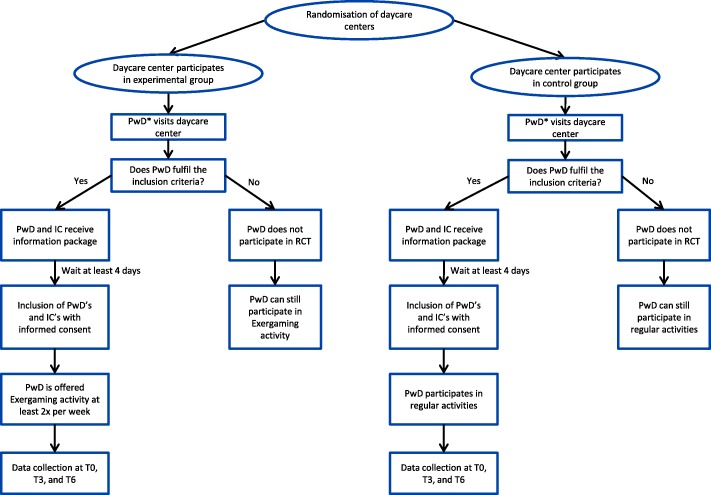
Flow chart of the recruitment and participant timeline

An overview of the recruitment and participant timeline can be found in Fig. 2. In this RCT, dyads will participate each consisting of a person living with dementia, who attends a day-care centre and his or her informal caregiver. Participants with dementia can be either existing or new visitors of a day-care centre in the Netherlands.

### Inclusion criteria

Inclusion criteria are as follows. The participant with dementia has to live in the community and not foreseen to be admitted in a care home in the coming six months. He or she has to visit the day-care centre for at least two days per week. If the participant is admitted to a nursing or care home, stops visiting the day-care centre during the six months of data collection, and/or deceases his/her participation in the trial will be withdrawn. There has to be a diagnosis of mild to moderate dementia (MMSE score between 10 and 24), without any restriction to the type of dementia (i.e. Alzheimer’s Disease, frontotemporal dementia, vascular dementia), or the authority, who has given the diagnosis (i.e. general practitioner, neurologist, memory clinic). There has to be an informal caregiver present, who is willing to participate in interviews and to keep track of the healthcare costs of themselves and the participant with dementia.

### Exclusion criteria

Exclusion criteria are as follows. Severe physical disorders or diseases, which would make it impossible for the participant with dementia to take part in an exergaming activity. Terminal diseases (other than dementia).

### Recruitment

Participants are recruited by the staff of the day-care centres. Each day-care centre has a local project coordinator (i.e. physiotherapist, activity coordinator) assigned among their staff. This person is instructed by the research team on how to recruit participants and supervise the intervention and data collection.

To inform potential participants the project coordinator at the day-care centres offers an information package to the person with dementia and their informal caregiver.

Informal caregivers are offered a small compensation (worth € 25,-) for their participation in the research project. The compensation is meant as an extra motivation for them to take part, especially because informal caregivers can often feel burdened which might refrain them from participating [49]. It is not compulsory to finish the entire data collection trajectory (i.e. all interviews and tests for both informal caregiver and participant with dementia) in order to receive the compensation.

### Sample size calculation

Since a high quality study evaluating the effect of a similar intervention on physical activity and mobility was not available when we were writing our protocol, we used previous results on exergaming regarding one of our secondary outcome measures (memory) for this power calculation [38]. We performed a power analysis based on a power of 80% and a difference of 2 (with a standard deviation of 4.6) between scores on the Mini-Mental State Examination (MMSE) [50]. We chose an Intracluster Correlation Coefficient (ICC) of 0.05. This resulted in a sample size of 166 participating dyads with an alpha of 0.05. When also taking into account the cluster effect of the day-care centres (7 dyads per cluster) and drop out (4%), the total sample size needed is 224 dyads.

### Intervention and materials

Participants with dementia visiting a day-care centre assigned to the experimental group will be asked to take part in the exergaming intervention, which consists of interactive cycling. This type of exergaming has been selected for this research study, because is it a recognizable physical activity, especially in the Netherlands where cycling is very common. During interactive cycling, the participant cycles on a stationary bicycle (i.e. Thera trainer or home trainer) that is connected to a digital screen. The participant can cycle a route and is thus physically and cognitively stimulated. It is also possible to place multiple bicycles in one room. The exergames vary in physical and cognitive challenges and level of interaction. Interactive cycling on a stationary bicycle is made possible by specific software and sensors: the participant’s cycling speed regulates the images on a digital screen. Motion sensors are attached to the pedals of the bicycle. Depending on the chosen software, specific interactions are possible, such as making left or right turns on junctions, or creating an own route using *Google Street View*©. The available routes vary per program, examples of routes are Amsterdam city centre, Rotterdam Blijdorp Zoo, the Betuwe (area in the Netherlands with lots of orchards), Rome, and Tokyo.

Day-care centres participating in the study already have the exergaming equipment or can buy or lease it from various providers. The companies Embedded Fitness and SilverFit participate in the research project as partners. Within the context of this research project, they offer participating day-care centres four different kinds of exergaming systems with a discount: “*Fietslabyrint*©”, “*SilverFit Mile*©”, “*PraxFit*©” or “*DiFiets*©”. The software, sensors and the digital screen can be connected to any kind of exercise equipment with a pedalling mechanism. This means that if a day-care centre already has a stationary bicycle, they do not need to purchase a new one. If this is not the case, SilverFit and Embedded Fitness sell a wide selection of stationary bicycles.

Because purchasing exergaming equipment can be costly, day-care centres in the experimental group each receive an extra allowance of € 1.500,-. The research team also provides them with a list of organizations where they can apply for additional funding.

The research team supports the day-care centres in choosing the exergaming equipment best suited to their needs in terms of type of stationary bicycle which fits the target group as well as the available facilities at the location. This is done based on the results of previous research carried out by the Coalition of Applied Gaming [35], the expertise of the research team and interviews with (representatives of) people with dementia from participating day-care centres. The research team will also advise with regard to the space where and manner in which the exergaming activity will be offered (appealing, accessible, bright space; possibilities to interact with other people).

Day-care centres in the experimental group also receive information resulting from the process evaluation alongside the trial. This will make it possible for them to optimize the manner in which they offer exergaming while the trial is still running. Additionally, they are offered a free training for their staff as well as specific instructions for their exergaming equipment. Day-care centres are advised to offer the exergaming intervention five times per week as a regular activity in their program. This is consistent with recommendations for physical activity for community-dwelling older people as well as those in residential care [51–54]. Research participants with dementia are encouraged to take part in the exergaming activity at least twice per week for the duration of the trial. Of course, they can participate in any other physical exercise or activities in addition the exergaming activity if they wish to do so.

### Control group: Regular activities

Participants with dementia in the day-care centres in the control group can join the regular activities. This can be a variety of recreational activities, as well as physical exercise activities such as gymnastics in a group setting, walking outdoors, etc. Day-care centres in the control group are also entitled to the same discount on the exergaming equipment and the list of optional funding organizations. However, they will not use the exergaming equipment yet for the duration of the trial.

### Outcome measures

#### Primary outcome measures

The primary outcome measures are the physical activity and mobility of the participants with dementia and can be found in Table 1. Physical activities (including exergaming) he/she is engaging in (how many times a week, and for how long) in the day-care centre and at home will be registered using a custom-developed registration form during 1 week at the three measuring moments. With regard to mobility, information will be gathered with a short physical test, the Short Physical Performances Battery (SPPB) [55]. This test consists of three subtests: balance, gait speed, and chair stand and is used to evaluate lower extremity functioning in older adults.

**Table 1:** Primary outcome measures for participants living with dementia

Outcome	Outcome measures for participants with dementia	Recorded by	Timing		
			Baseline	3 months	6 months
Mobility	Short Physical Performance Battery (SPPB) [55]	Researcher by means of test with participant with dementia	X	X	X
Physical activities of the participant living with dementia	Custom-developed registration form	Informal caregiver	X	X	X
Physical activities of the participant living with dementia	Custom-developed registration form	Staff at day-care centre	X	X	X

#### Secondary outcome measures

##### Economic evaluation

The economic evaluation will be performed from a societal perspective. Healthcare utilization and informal care will be measured using cost diaries, and costs will be calculated according to the Dutch guidelines for economic evaluations [56]. Costs of the exergaming intervention are calculated using a bottom-up approach based on information with interviews with staff members at the day-care centre using the form ‘Costs and benefits of applied games’ [35]. Quality of life will be measured using the three-level version of the EuroQol questionnaire (EQ-5D-5 L) [57]. EQ-5D-5 L health states will be converted to utility scores using the Dutch EQ-5D-5 L tariff [58]. The utility scores will be used to calculate Quality-Adjusted Life-Years (QALYs) using the area-under-the-curve method. The secondary outcomes can be found in Tables 2, 3, and 4.

**Table 2:** Secondary outcome measures for participants living with dementia

Outcome	Outcome measure	Recorded by	Timing		
			Baseline	3 months	6 months
Physical functioning	Physical Activity Scale of the Elderly (PASE) [67]	Researcher during interview	X	X	X
Cognitive functioning	Mini-Mental State Examination (MMSE) [68, 69]	Researcher by means of test	X	X	X
Cognitive functioning	Trail Making Test (TMT) [70]	Researcher by means of test	X	X	X
Social functioning	One question from the Adult Social Care Outcomes Toolkit (ASCOT) [71]	Researcher during interview	X	X	X
- Demographics and personal characteristics - Physical functioning - Cognitive functioning - Social functioning - Emotional functioning - Quality of life	The Older Persons and Informal Caregivers Survey Minimum DataSet (TOPICS-MDS care recipient) [72], including EuroQol five dimensions questionnaire with five-level scale (EQ-5D-5 L) [73, 74]	Researcher during interview	X	X	X
Interest in and enjoyment of physical exercise	Intrinsic Motivation Inventory (IMI) [66]	Researcher during interview	X	X	X
Satisfaction with the exergaming activity (*only experimental group*)	Separate questions	Researcher during interview	X	X	X
Body Mass Index (BMI)	Weight scale and measuring tape to measure height	Researcher during interview	X

**Table 3:** Secondary outcome measures for informal caregivers

Outcome	Outcome measure	Recorded by	Timing			
			Baseline	3 months	6 months	0–6 months (ongoing)
Physical functioning of the participant living with dementia	Physical Activity Scale of the Elderly (PASE) [67]	Researcher during interview	X	X	X	
Fall incident rate of the participant with dementia during the past 3 months	Custom-developed registration form	Informal caregiver	X	X	X	
- Behaviour and mood of the participant living with dementia - Emotional burden for the informal caregiver	Neuropsychiatric Inventory-Questionnaire (NPI-Q) [75, 76]	Researcher during interview	X	X	X	
For both informal caregiver and participant with dementia: - Demographics and personal characteristics - Physical functioning - Cognitive functioning - Social functioning - Emotional functioning - Quality of life - Healthcare costs Experiences of the informal caregiver	The Older Persons and Informal Caregivers Survey Minimum DataSet (TOPICS-MDS informal caregiver) including EuroQol five dimensions questionnaire with five-level scale (EQ-5D-5 L) [72–74]	Researcher during interview	X	X	X	
Subjective burden for the informal caregiver	Short Sense of Competence Questionnaire (SSCQ) [77]	Researcher during interview	X	X	X	
Positive care experiences of the informal caregiver	Scale of Positive Experiences (Positieve Ervaringen Schaal (PES)) [78]	Researcher during interview	X	X	X	
- Healthcare costs - Unexpected (Adverse) Events, falls and reasons of (potential) drop out	(Care) diaries (one for the participant living with dementia, one for the informal caregiver)	Informal caregiver				X
Experience of the participant living with dementia with sports/cycling, technology and digital games in the past	Separate questions	Researcher during interview	X

**Table 4:** Secondary outcome measures for staff at day-care centres

Outcome	Outcome measure	Recorded by	Timing		
			Baseline	3 months	6 months
- Fall incident rate of the participant with dementia during the past 3 months - Social context and extent of supervision during the exergaming activity (*only* experimental group)	Custom-developed registration form	Staff at day care centre	X	X	X
Social functioning of the participant living with dementia in the day care centre	GIP: Behaviour Observation Scale for Intramural Psychogeriatrics: sub scale 1 (Gedragsobservatieschaal voor de Intramurale Psychogeriatrie (GIP): subschaal 1) [79]	Staff at day care centre	X	X	X
Cost effectiveness	Fill out form ‘Costs and benefits of applied games’ (Invulformulier ‘Kosten en opbrengsten van applied games’) [35]	Researcher during interview (with staff of day care centre, *only experimental group*)			X
Implementation aspects of exergaming (*only experimental group*)	Measurement Instrument for Determinants of Innovations (MIDI) (MIDI: Meetinstrument Determinanten van Innovaties) [59, 60]	Online survey for staff of day care centre	X		X

##### Process evaluation

For the exploration of implementation aspects of exergaming the Measurement Instrument for Determinants of Innovations (MIDI) [59, 60] will be applied. The MIDI is survey that measures 29 items, scored on a 5-point Likert scale, covering determinants of innovations that may affect its implementation, along four domains: the intervention itself, in this case the exergame (e.g., the exergame is perceived useful), the user, in this case the professional at the day-care centre that will apply the exergame (e.g., the professional feels sufficiently skilled to operate the exergame), the organization (e.g., there is enough time available to operate exergame), and the socio-political context (e.g., the patient is motivated to use the exergame). Several MIDI items will be adapted for this trial to fit the anticipated goals, benefits and downfalls, and stakeholders involved, based on short interviews with the participating organizations. Also, strategies applied to support the implementation (e.g., organize instruction meetings or develop instruction materials) are surveyed. Data will also be gathered on the characteristics of the respondent (age, gender, discipline), type of exergame applied in the study, and level of experience with use and implementation of exergames.

The MIDI will be administered among staff of the day-care centres in the experimental group. Based on the results, these organizations will receive recommendations on how to enhance implementation, which are tailored to their specific situation and circumstances. Towards the end of the trial, they will be asked to fill this survey out again.

Based on these results of both survey rounds, qualitative in depth interviews (maximum amount will be four) with day-care centres will be conducted. That is to say, with the centres that implemented the exergaming intervention most successfully (i.e., who scored the highest on implementation determinants over time and/or report the highest usage of the exergame) and those, who implemented exergaming least successfully (i.e., who scored the lowest on implementation determinants over time and/or report the highest usage of the exergame). During the interview we will discuss what the most influential determinants are for the implementation of the exergame and which strategies can be best applied to cope with ‘barriers’ and make use of ‘drivers’ (factors which enhance implementation). At the end of the trial, we will organise focus groups with the persons involved (participants with dementia, informal caregivers, staff of day-care centres and exergame providers) to measure the satisfaction with the physical activities offered in the day-care centres and to discuss future possibilities with regard to optimizing implementation.

### Procedure and ethics

The protocol, procedure and informed consent of this research study are approved by the Medical Ethics Committee (METc) of the Amsterdam University Medical Centers (UMC), location VU University medical center (VUmc) (file number NL58227.029.16). Any amendments to the protocol will also be submitted to the METc for approval, and sent to the NTR. Participation is voluntary for the day-care centres, participants with dementia and their informal caregivers, and they can stop participation at any time without having to explain their reasons. All participating day-care centres will sign a declaration of participation. All participants with dementia and their informal caregivers will sign an informed consent form. Additionally, the consent form for the participant with dementia also will be signed by the informal caregiver or legal representative.

After informed consent has been obtained, interviews and tests will be done involving the participants with dementia and their informal caregivers on three different occasions: at the start, after three and 6 months. To reduce any bias resulting from the sequence of the outcome measures, the order of the outcome measures used during the interviews will be partly randomized for each interview. Trained researchers will interview and test the participants with dementia in the day-care centres. Informal caregivers will be interviewed by telephone, unless an informal caregiver prefers to be interviewed in person. During these 6 months, the informal caregivers will keep two care diaries. In these, they will register their own use of health care facilities and medicines, as well as that of the participant with dementia.

### Blinding

Due to the design of the study and the nature of the intervention, blinding of staff of day-care centres, participants with dementia and informal caregivers is not possible. Because there are a few different questions in the outcomes measures for the control and experimental group (i.e. questions about the exergaming intervention) interviewers could also not be blinded.

### Data management

Data management will be conducted according to principles of International Conference on Harmonisation of technical requirements for registration of pharmaceuticals for human use/ Good Clinical Practice [61]. Data will be coded and entered into a Castor [62] database specifically built for this trial, and which is only accessible for the research team. Five percent of the data entry will be checked to see if the rate of errors is below the range of 3%. If not, all data will be double entered and checked. Range checks for data values will be done to account for unexpected or erroneous outliers. The identification codes of the research participants consist of a number for the day-care centre they are visiting and a number for the participant with dementia/informal caregiver. The list of codes with names of participants with dementia and informal caregivers is stored in a separate administrative Access database, which is only accessible for members of the research team. The (coded) digital research data are stored for at least 15 years. The paper data from the participants will be kept until 5 years after publication and then destroyed. Data monitoring will be arranged through the METc of the Amsterdam UMC, location VUmc, to whom the research team will provide an annual report of the study.

### Project group and advisory committee

The project group has 12 members from different organizations and areas of expertise (eg, academic researchers from Amsterdam UMC and Vrije Universiteit Amsterdam, a physiotherapist from HilverZorg, a large care organization). This group meets every 3 months. The project also has an Advisory Committee, consisting of seven members, who meet every 6 months. Both groups discuss the ongoing research project, including any issues that may arise and solutions to contribute to promote continuation of the trial and dissemination strategies. Whereas members of the project group execute the research project, the Advisory Committee has an advising role.

### Analysis of the data

#### Effect evaluation

Descriptive statistics at baseline will be used to compare participants with dementia, informal caregivers and staff of day-care centres in the experimental group with the control group. This will provide information on the differences between the intervention and control group at the start of the study.

The baseline scores of the people, who drop out of the study will be compared to those, who remain in the study. This will provide information about selective drop-out. Participants, who drop out during the trial will be included in the main analysis by imputing their scores using multiple imputation.

The effectiveness of the intervention will first be examined by comparing percentages, or mean scores (Chi-Square test, Mann-Whitney U test and T-tests (*p* < 0.05). Second, we will use mixed model regression analysis which will take into account the effect of clustering on the level of the day-care canters, confounders and missing data. The regression analysis will be conducted in three steps. Firstly, an analysis will be carried out for the primary and secondary outcome measures. If any differences in variables related to the outcome measures between the experimental group and the control group are found at baseline, these are added as potential confounders to the model for each outcome measure to check whether the uncorrected Beta-coefficient (β) changes with more than 10%, which means that this variable will remain in the model as a confounder. Examples of potential confounders are type of exergaming equipment, amount of support from staff, severity of dementia etc. Finally, mixed model analysis will be conducted for each outcomes variable for both follow up measurements (three and 6 months) with the selected confounders included in the analysis.

The method of QUalitative INteraction Trees (QUINT) [63] will be used for subgroup analysis. This allows for the identification of subgroups for whom the effect of the intervention differs, for example differences related gender, age, ethnicity or severity of dementia. Cut-off values do not need to be defined beforehand. Based on the QUINT analysis, more specific advice can be formulated with regard to which type of participant or informal caregiver the intervention has a positive effect or not.

#### Process evaluation

For the day-care centres in the intervention group, after the first survey, we will analyse the scores on determinants of implementation within the centre and between the centres. Based on the results, we will assess how well these centres score on determinants and we will draft recommendations on how, i.e., exemplary strategies, to address determinants, which score 3 or lower. After the second survey, we will compare scores between the centres. We will assess which centre overall scores highest and/or made the largest improvement in scores on determinants for implementation. Also, we will assess which centre overall scores the lowest on determinants for implementation. Moreover, we will combine this data with data on self-reported use of the exergames by patients, to select two to four centres to interview. Notes of the in-depth interview will be made. Audio recordings will be made of the conversations during the focus groups. These tapes will be transcribed verbatim and these transcripts will be thematically coded regarding factors influencing the implementation of exergaming independently by two members of the research team and then summarized.

#### Economic evaluation

The primary outcome in the economic evaluation will be QALYs. In addition, the economic evaluation will be done for the primary outcome measures of the trial: physical activity and physical functioning using the (SPPB) [55]. Bivariate regression analyses will be done to estimate the differences in costs and effects between the groups. If necessary, costs and effects will be adjusted for confounders. Incremental cost-effectiveness ratios (ICERs) will be calculated by dividing the difference in total costs between the two groups by the difference in effect. Bias-corrected accelerated bootstrapping with 5000 samples will be used to estimate statistical uncertainty. The bootstrapped cost-effect-pairs will be plotted in a CE plane and ‘cost-effectiveness acceptability curves’ will be estimated.

### Harms

#### Risk/benefit assessment

The risk of harm when participating in the exergaming intervention is in our opinion negligible, and comparable to the risk of participating in regular physical exercise activities (or lower, as this is in a safe environment). Although physical injuries cannot be ruled out, we will try to prevent them as much as possible. During the exergaming intervention, a member of staff of the daycare center will be present to supervise. If a daycare center doesn’t have exergaming equipment yet, they are advised about the purchase of the exergaming equipment for people living with dementia and are offered a training in the use of this equipment.

We think the (results of the) research project can have benefits for people living with dementia and their informal caregivers. We hope that, if exergaming proves to yield positive effects, this will result in the intervention becoming available for more people living with dementia. To measure the effects, it is essential to let potential end users themselves try out and evaluate the exergames, and therefore involve them in the research project. In our opinion, the potential benefits of the research project are in balance with the potential risk and burden for people with dementia participating in the trial.

#### (serious) adverse events

Adverse events (AE’s) and serious adverse events (SAE’s) are specified according to definitions the Central Committee on Research Involving Human Subjects (CCMO) [64]. They will be registered and reported to the METc of the Amsterdam UMC, location VUmc / CCMO following the procedure which is described on the CCMO website [64]. All (S)AE’s will be monitored, until the risk of harm is gone or until a stable (end) state has been reached. Depending on the situation, extra tests might be necessary, or a referral to a doctor. The research team will keep an up-to-date overview of all (S)AE’s until the end of the trial.

### Trial status

Recruitment of day-care centres commenced July 2016 and ended in August 2018. Inclusion of participants within the day care centres started with the first interview taking place in February 2017 and was finished in September 2018. At the time of the initial submission of the manuscript, data-collection was still ongoing and should be finished by March 2019. For the process evaluation, the first MIDI list was sent out to day-care centres in the experimental group in July 2017 and the second one in December 2018, and the qualitative interviews regarding implementation issues will take place in the first quarter of 2019. Focus groups are planned to take place in February/March 2019.

### Dissemination policy

Trial results will be communicated to several different target groups: people living with dementia, older people, informal caregivers, volunteers, healthcare professionals (staff of day-care centres, meeting centres, nursing homes), policy makers, managers of care organisations, insurance companies, researchers from various backgrounds (dementia care, game design, movement sciences, psychology, physiotherapy, geriatric medicine and general practice), and companies developing and selling exergames, Each target group will be reached using appropriate communication channels, eg lectures and presentations at different kinds of conferences/meetings, digital newsletters, websites, social media, networks, (scientific) journals, and magazines. Authorship will only be granted to those who contribute to writing a publication. No professional writers will be used for publications, except for language checks in non-Dutch articles.

## Discussion

Large and scientifically robust studies to evaluate the effects of exergaming for people living with dementia are lacking. The aim of this study is to evaluate the effectiveness and cost-effectiveness of exergaming for people living with dementia and their informal caregivers. If the exergaming intervention proves to have positive effects, this may boost the uptake of the exergaming intervention in other day-care centres. Implementation will be supported by the findings from our process evaluation, which will provide extensive information on factors that either promote or hinder successful implementation of exergaming for this target group.

We expect that exergaming will have positive effects on people living with dementia in several domains such as physical, cognitive, emotional, and social functioning, and on quality of life for both people living with dementia and their informal caregivers. We hope that the results of this project will motivate people with dementia to exercise more and as a result to experience positive effects on their wellbeing and the quality of life. We envision that this may have a positive effect on the burden of care experienced by their informal caregiver as well. Finally, we expect that societal costs of the people living with dementia and their informal caregivers are affected.

There are several challenges that lie ahead when starting this research project. Recruitment can be a complicated process with several steps that have to be taken. Firstly, day-care centres have to be found that are willing to participate and can have funding ready in case they are assigned to the experimental group. Secondly, the research team has to effectively collaborate with the local project coordinators of the day-care centres. Successful recruitment can very much depend on this collaboration as well as whether the local coordinator is able to motivate visitors of his/her day-care centre (and their informal caregivers) to participate in the trial. We will support the staff of day-care centres as much as possible in the process of recruitment, for example by written instructions for the local coordinator of the day-care centre regarding recruitment and motivating participants.

There have been several budget cuts in the healthcare sector in the Netherlands, which puts time pressure on staff of day-care centres. This can negatively impact both recruitment of day-care centres and participants with dementia for our trial. However, our research project partly reimburses day-care centres (both in the experimental and control group) to participate in the trial. In addition, we negotiated a discount on exergaming equipment for them and a list of organizations where they can apply for additional external funding. Finally, staff of participating day-care centres get the opportunity to attend a free training afternoon on how to use the exergaming equipment and to engage in an innovative technology-based intervention.

For day-care centres in the control group, recruitment of participants might be more difficult. Because participants with dementia will not get the intervention, the importance and potential benefit of participating in the study might be less clear to them (and to the informal caregivers). This could make it more challenging for the local coordinator to explain and motivate them to participate. In both the experimental and control group, some informal caregivers might already feel overburdened, and thus might not be willing to participate in the study, because they overestimate the time it will cost them.

With regard to the outcome measures, it is a challenge in the field of assistive technology to select appropriate outcome measures [65]. In our study, some outcome measures may be complicated to assess among people living with dementia. For example, the Intrinsic Motivation Inventory (IMI) [66] consists of a long list of statements and has a seven point scale for answering, which can be confusing. To mitigate this problem, we will use a printed card with the answering scale. Also, certain outcome measures can be challenging if people are deaf, analphabetic or not native Dutch speakers. However, this was not taken into account when we established our in- and exclusion criteria. Furthermore, because of the number of outcome measures, the duration of the interviews can be quite long, especially for people living with dementia, but also for informal caregivers, who are already very busy. To minimize the occurrence of problems during the interviews, interviewers receive a training with extensive instructions. For example, they are instructed on how to assess each outcome measure, but also to look out for and deal with distress during the interview. If needed, the interview can be divided into two shorter interviews during two subsequent appointments instead of one.

Next to the interviews with people living with dementia, we will collect data from the informal caregivers and the staff of day-care centres. This method of collecting data is prone to errors and could easily be forgotten by those involved. To minimize those problems the research team will remind the staff of day-care centres and informal caregivers frequently.

Another limitation of this research project is that merely one type of exergaming is included. Therefore, the research findings can be typical for only interactive cycling and it is not possible to be sure that findings are generalizable among other types of exergames.

This research project is part of the Marie Curie Innovative Training Network (ITN) Interdisciplinary Network for Dementia Using Current Technology (INDUCT). This ITN offers many different perspectives and insights. Various academic research areas are represented, such as psychology, medicine, occupational therapy, nursing, movement sciences, and anthropology. Additionally, people working outside of academia are also part of the network, for example from commercial industry or care organizations. Moreover, both our exergaming research project group and advisory group meet regularly to offer their multidisciplinary input. In terms of patient and public involvement (PPI), an former informal caregiver is part of our project group, and the European Working Group of People with Dementia (EWGPWD) is consulted several times.

In summary, this study will contribute to the evidence base on the effectiveness and cost effectiveness of exergaming for people living with dementia and their informal caregivers. It will also yield information about implementation of exergaming for this target group. If this study demonstrates positive results on physical, cognitive, emotional, and/or social functioning in people living with dementia and on quality of life of people living with dementia and their informal caregivers, this will hopefully contribute to exergaming becoming available for more people living with dementia.

## Additional file

Additional file 1: WHO_Trial_Registration_Data_Set_Van Santen_2019.pdf (PDF 84 kb)

## Abbreviations

ASCOT: Adult social care outcomes toolkit
BMI: Body Mass Index
CE: Cost-effectiveness
CONSORT: Consolidation Standards of Reporting Trials
EQ-5D-5 L: EuroQol five dimensions questionnaire with five-level scale
EWGPWD: European Working Group of People with Dementia
GIP: Gedragsobservatieschaal voor de Intramurale Psychogeriatrie (Behaviour Observation Scale for Intramural Psychogeriatrics)
ICC: Intracluster Correlation Coefficient
ICER: Incremental cost-effectiveness ratios
IMI: Intrinsic Motivation Inventory
INDUCT: Interdisciplinary Network for Dementia Using Current Technology
ITN: Innovative Training Network
METc: Medisch ethische toetsingscommissie (medical ethics committee)
MIDI: Measurement Instrument for Determinants of Innovations
MMSE: Mini-Mental State Examination
NPI-Q: Neuropsychiatric Inventory-Questionnaire
NTR: Netherlands Trial Register
PASE: Physical Activity Scale of the Elderly
PES: Positieve ervaringen schaal (scale of positive experiences)
PPI: Patient and public involvement
QALYs: Quality-Adjusted Life-Years
QUINT: QUalitative INteraction Trees
RCT: Randomized Controlled Trial
SPIRIT: Standard Protocol Items: Recommendations for Interventional Trials
SPPB: Short Physical Performance Battery
SSCQ: Short Sense of Competence Questionnaire
TMT: Trail Making Test
TOPICS-MDS: The Older Persons and Informal Caregivers Survey Minimum DataSet
UMC: University Medical Centers
VUmc: VU University medical center
