# Supplementary material for: The (cost-) effectiveness of exergaming in people living with dementia and their informal caregivers: protocol for a randomized controlled trial
Source: BMC Geriatr. 2019 Feb 19;19:50. doi: 10.1186/s12877-019-1062-x (PMC6381728; doi:10.1186/s12877-019-1062-x)
Supplement: Supplementary file 1 — WHO_Trial_Registration_Data_Set_Van Santen_2019.pdf (PDF 84 kb) [file 12877_2019_1062_MOESM1_ESM.pdf]

search OK

Home

Who are we?

Why register?

Signup for registration

Online registration

Log in to register your trial

Search a trial

NRT en CCMO

Contact

NEDERLANDS

MetaRegister van CCT (UK)

ISRCTN-Register van CCT (UK)

TRIAL INFO

Improvement of engagement in physical activities and quality of life in dementia: study into the (cost-)effectiveness of exergaming

- CANDIDATE NUMBER	23477
- **NTR NUMBER**	**NTR5537**
- ISRCTN	ISRCTN no longer applicable
- DATE ISRCTN CREATED	
- DATE ISRCTN REQUESTED	
- DATE REGISTERED NTR	10-dec-2015
- SECONDARY IDS	ZonMw 70 73308-98 CMG: VUmc2015-679
- PUBLIC TITLE	Improvement of engagement in physical activities and quality of life in dementia: study into the (cost-)effectiveness of exergaming
- SCIENTIFIC TITLE	(Cost-)effectiveness of exergaming in dementia
- ACRONYM	D-EXERGAMING
- HYPOTHESIS	Persons with dementia who engage in exergaming in day centres will be more physically active and show improved functioning compared to day centres offering regular activities.
- HEALT CONDITION(S) OR PROBLEM(S) STUDIED	Dementia, Informal care-givers, Physical activity, Quality of life
- INCLUSION CRITERIA	diagnosis of dementia, severity mild to moderate severe<br>living in the community<br>attending day care<br>primary caregiver involved
- EXCLUSION CRITERIA	admission to nursing home expected within 6 months<br>terminal illness<br>severe physical comorbidity
- MEC APPROVAL RECEIVED	yes
- MULTICENTER TRIAL	yes
- RANDOMISED	yes
- MASKING/BLINDING	None
- CONTROL	Active
- GROUP	Parallel
- TYPE	2 or more arms, randomized
- STUDYTYPE	intervention
- PLANNED STARTDATE	1-mrt-2016
- PLANNED CLOSINGDATE	31-mrt-2019
- TARGET NUMBER OF PARTICIPANTS	224
- INTERVENTIONS	Exergaming (doing physical exercises in a virtual cognitive gaming environment) as compared to regular activities in day care
- PRIMARY OUTCOME	Physical activity (lichamelijke activiteit)
- SECONDARY OUTCOME	Physical functioning (lichamelijk functoneren)<br>Cognitief functioning (cognitief functioneren)<br>Social functioning (sociaal functioneren)<br>Behaviour and mood (gedrag en stemming)<br>Quality of life (kwaliteit van leven)<br>Experienced burden (ervaren belasting mantelzorgers)
- TIMEPOINTS	at baseline, and three and six months later
- TRIAL WEB SITE	not yet available
- STATUS	open: patient inclusion
- CONTACT FOR PUBLIC QUERIES	Dr. Franka Meiland
- CONTACT FOR SCIENTIFIC QUERIES	Dr. Franka Meiland
- SPONSOR/INITIATOR	VU University Medical Center, Vrije Universiteit Amsterdam, TNO, Hilverzorg, Evean, Embedded fitness, Alzheimer afd Amsterdam, Silverfit
- FUNDING (SOURCE(S) OF MONETARY OR MATERIAL SUPPORT)	ZON-MW, The Netherlands Organization for Health Research and Development, EU grant
- PUBLICATIONS	van Santen J, Dröes R-M, Holstege M, Blanson Henkemans OA, van Rijn A, de Vries R, et al. Effects of Exergaming in People with Dementia: Results of a Systematic Literature Review. Journal of Alzheimer's Disease. 2018;64(2):741-60.
- BRIEF SUMMARY	In this project, the (cost-)effectiveness of an innovative way of exercising for people with dementia will be studied: exergaming. Exergaming technology focuses on gaming

exercises that are controlled by physical movements of the player. The aim of this intervention is to stimulate people living with dementia to exercise indoors in an enjoyable, safe way, thereby promoting physical, cognitive, emotional and social functioning and quality of life. A randomized controlled trial will be conducted among participants with dementia who attend day-care centers with or without exergaming activities. The effectiveness of exergaming will be compared to regular activities in day-care centers on performance of physical activities and mobility (primary outcome) and on physical, cognitive, social and emotional functioning, and quality of life of people with dementia. For informal caregivers, the effectiveness will be evaluated on experienced burden, quality of life and positive care experiences (secondary outcomes). Furthermore, the cost-effectiveness will be studied as well as the satisfaction with exergaming and factors to improve implementation of exergaming in dementia care.
Participants will be recruited from the Netherlands.

- MAIN CHANGES (AUDIT TRAIL) 28-dec-2018 -IK:

PRIMARY OUTCOME:
"Physical activity (lichamelijke activiteit)"

replaced by

"Physical activity (lichamelijke activiteit) en Mobility (mobiliteit)"

SECONDARY OUTCOME:
"Physical functioning (lichamelijk functoneren)
Cognitief functioning (cognitief functioneren)
Social functioning (sociaal functioneren)
Behaviour and mood (gedrag en stemming)
Quality of life (kwaliteit van leven)
Experienced burden (ervaren belasting mantelzorgers)"

replaced by

"For the participant with dementia:
Physical functioning & fall incident rate (lichamelijk functioneren & valincidenten)
Cognitief functioning (cognitief functioneren)
Social functioning (sociaal functioneren)
Emotional functioning (emotioneel functioneren)
Healthcare costs (gezondheidsgerelateerde kosten)
Body Mass Index (BMI)
Quality of life (kwaliteit van leven)
Experience with sports/cycling, technology and digital games in the past (ervaring met sport/fietsen, technologie en computerspellen in het verleden)
Unexpected (Adverse) Events (onverwachte nadelige gebeurtenissen)
Interest in and enjoyment of physical exercise (interesse in en motivatie voor beweegactiviteiten)
Satisfaction with the exergaming activity (only experimental group) (tevredenheid met de exergaming activiteit, alleen in experimentele groep)
Social context and extent of supervision during the exergaming activity (only experimental group) (sociale context en mate van toezicht tijdens de exergaming activiteit, alleen in experimentele groep)
Demographics and personal characteristics (demografische en persoonlijke gegevens)

For the informal caregiver:
Experienced burden (ervaren belasting mantelzorgers)
Quality of life (kwaliteit van leven)
Positive care experiences (positieve ervaringen van het mantelzorgen)
Healthcare costs (gezondheidsgerelateerde kosten)
Unexpected (Adverse) Events (onverwachte nadelige gebeurtenissen)
Demographics and personal characteristics (demografische en persoonlijke gegevens)

For the day-care centers (only experimental group:
Cost effectiveness (kosteneffectiviteit)
Implementation aspects of exergaming (only for experimental group) (implementatie-aspecten van exergaming, alleen voor experimentele groep)"

- RECORD 10-dec-2015 - 28-dec-2018

- Indien u gegevens wilt toevoegen of veranderen, kunt u een mail sturen naar **nederlands@trialregister.nl**
